# The Adaptor Protein Swiprosin-1/EFhd2 Is Dispensable for Platelet Function in Mice

**DOI:** 10.1371/journal.pone.0107139

**Published:** 2014-09-22

**Authors:** Martina Morowski, Sebastian Brachs, Dirk Mielenz, Bernhard Nieswandt, Sebastian Dütting

**Affiliations:** 1 University of Würzburg, Department of Experimental Biomedicine, University Hospital and Rudolf Virchow Center for Experimental Biomedicine, Würzburg, Germany; 2 Division of Molecular Immunology, Department of Internal Medicine III, Nikolaus Fiebiger Center, University Hospital Erlangen and University of Erlangen-Nürnberg, Erlangen, Germany; King's College London School of Medicine, United Kingdom

## Abstract

**Background:**

Platelets are anuclear cell fragments derived from bone marrow megakaryocytes that safeguard vascular integrity, but may also cause pathological vessel occlusion. Reorganizations of the platelet cytoskeleton and agonist-induced intracellular Ca^2+^-mobilization are crucial for platelet hemostatic function. EF-hand domain containing 2 (EFhd2, Swiprosin-1) is a Ca^2+^-binding cytoskeletal adaptor protein involved in actin remodeling in different cell types, but its function in platelets is unknown.

**Objective:**

Based on the described functions of EFhd2 in immune cells, we tested the hypothesis that EFhd2 is a crucial adaptor protein for platelet function acting as a regulator of Ca^2+^-mobilization and cytoskeletal rearrangements.

**Methods and Results:**

We generated EFhd2-deficient mice and analyzed their platelets *in vitro* and *in vivo*. *Efhd2^-/-^* mice displayed normal platelet count and size, exhibited an unaltered *in vivo* life span and showed normal Ca^2+^-mobilization and activation/aggregation responses to classic agonists. Interestingly, upon stimulation of the immunoreceptor tyrosine-based activation motif-coupled receptor glycoprotein (GP) VI, *Efhd2^-/-^* platelets showed a slightly increased coagulant activity. Furthermore, absence of EFhd2 had no significant impact on integrin-mediated clot retraction, actomyosin rearrangements and spreading of activated platelets on fibrinogen. *In vivo* EFhd2-deficiency resulted in unaltered hemostatic function and unaffected arterial thrombus formation.

**Conclusion:**

These results show that EFhd2 is not essential for platelet function in mice indicating that other cytoskeletal adaptors may functionally compensate its loss.

## Introduction

Platelets are small anucleated cell fragments derived from the cytoplasm of bone marrow megakaryocytes (MKs). At sites of vascular injury, platelets adhere and aggregate on the exposed subendothelial extracellular matrix (ECM) and thereby form a plug that seals the wound. This process is essential for normal hemostasis, but in diseased vessels it may lead to pathological thrombus formation and infarction of vital organs [1], [2]. Platelet activation on the injured vessel wall is induced by multiple signaling pathways and leads to extensive cytoskeletal rearrangements that are crucial for conversion from discoid to spheric shape, granule secretion, spreading and, at later time points clot retraction [3]. Many of these processes are controlled by proteins of the Rho family of small GTPases, like RhoA, Rac1, and Cdc42 and their downstream effector molecules such as the Wiskott-Aldrich syndrome proteins (WASPs), formins and p21-activated kinases (PAKs) [4]. In addition, cytoskeletal adaptor proteins such as talin1 and kindlin-3 are crucial for platelet function by linking the actin cytoskeleton to integrins thereby enabling their shift from low to high affinity for their ligands and thus stable platelet adhesion and aggregation [5], [6].

Elevation of cytosolic Ca^2+^ is a central step during platelet activation. It is induced through the release of Ca^2+^ from intracellular stores and subsequent Ca^2+^ entry through the plasma membrane, a process called store-operated Ca^2+^ entry (SOCE), which represents the major influx pathway of extracellular Ca^2+^ in platelets [7]. This process is regulated by the endoplasmic reticulum membrane-bound protein stromal interaction molecule 1 (STIM1) that senses Ca^2+^ depletion in intracellular stores via its EF hand motifs and Orai1, the major SOCE channel in the plasma membrane [7]. Finally, increased intracellular Ca^2+^ leads to the activation of the cytosolic EF-hand domain containing Ca^2+^ sensor CalDAG-GEFI [8] that links Ca^2+^ mobilization to signaling pathways regulating thromboxane A_2_ (TxA_2_) generation, integrin activation and granule release [9], thereby promoting platelet spreading [10], clot retraction [11], coagulant activity [12], [13], and platelet aggregation [14].

EF-hand domain containing 2 (EFhd2, also called Swiprosin-1) belongs to the EF-hand superfamily of Ca^2+^-binding proteins [15], consisting of an N-terminal region of low complexity with an alanine stretch, a functional SH3 binding motif [16], two functional EF hands [17] and a C-terminal coiled-coil domain [15], the latter capable of mediating self-oligomerization in a Ca^2+^-dependent manner [18]. Constitutive EFhd2-deficient mice revealed a function of this cytoskeletal adaptor protein in the negative regulation of germinal center-dependent humoral immunity [19]. Besides B cells and other hematopoietic cells [15], EFhd2 is predominantly expressed in the brain [20] and has been associated with tau-mediated neurodegeneration [21]. In addition, EFhd2 negatively regulates axonal transport in hippocampal neurons by inhibiting kinesin mediated microtubule gliding [22]. In natural killer (NK)-like cells, EFhd2 was found in the cytoskeleton fraction together with actin and actin-binding proteins such as α-actinin and filamin [23] and it was shown that EFhd2 directly binds to F-actin thereby modulating F-actin bundling and cell spreading in a Ca^2+^-dependent manner [18]. In line with this, EFhd2 co-localizes with F-actin and regulates actin remodeling in a human mast cell line [24], [25] and modulates lamellipodial dynamics by regulating the accessibility of F-actin to cofilin in melanoma cells [26]. A study characterizing sepsis-induced changes in the MK–platelet transcriptional axis revealed that EFhd2 mRNA was highly up-regulated in mice after induction of sepsis by the cecal ligation and puncture model [27]. Furthermore, a proteomic approach identified a minor up-regulation of EFhd2 expression in rat and human platelets upon stimulation with thrombin [28]. However, due to the lack of an appropriate animal model, the role of EFhd2 in platelet function has remained elusive.

Here we show that constitutive *Efhd2^-/-^* mice display unaltered platelet production and the function of the cells was indistinguishable from WT controls in a wide range of *in vitro* and *in vivo* assays suggesting that EFhd2 is not required for normal platelet function.

## Materials and Methods

### Mice

*Efhd2^-/-^* mice [19] and their wild-type littermates were on a C57Bl/6 background. Mice used for the experiments were between 6 and 12 weeks of age if not indicated differently. Animal studies were approved by the district government of Lower Franconia (Bezirksregierung Unterfranken).

### Chemicals and reagents

Midazolam (Roche Pharma AG), fentanyl (Janssen-Cilag GmbH) and the antagonists atipamezol (Pfizer), flumazenil and naloxone (both from Delta Select GmbH) were used according to the regulation of the local authorities. Thrombin (Roche Diagnostics), ADP, low-molecular-weight heparin and human fibrinogen (Sigma-Aldrich), U46619 (Enzo Life Sciences), collagen (Kollagenreagent Horm; Nycomed), apyrase Type III (Sigma-Aldrich), Fura-2 AM and Pluronic F-127 (Molecular Probes) were purchased, collagen-related peptide (CRP) was generated as described [29]. Antibodies against Rac1 (Millipore), actin (Sigma-Aldrich), tubulin (Millipore), mDia1 (Abcam), PAK1/2/3, phosphor-PAK1/2 (423/402), Syk, phosphor-Syk (Y519/520), cofilin, phospho-cofilin (Ser3) as well goat anti-rabbit IgG-HRP (all from Cell Signaling) were purchased. The antibody against the activated form of integrin αIIbβ3 (JON/A-PE) was from Emfret Analytics (Eibelstadt, Germany). Annexin-V was generously provided by Jonathan F Tait, University of Washington Medical Center and conjugated to DyLight 488 by standard methods. All other antibodies were generated and modified in our laboratories as previously described [30], [31].

### Platelet preparation

Mice were bled under isofluran anesthesia from the retro-orbital plexus. Blood was collected in a tube containing 20 U/mL heparin, and platelet-rich plasma (prp) was obtained by two cycles of centrifugation at 300 g for 6 minutes at room temperature (RT). For preparation of washed platelets, prp was washed twice at 800 g for 5 minutes at RT and the pellet was resuspended in Tyrodes-HEPES in the presence of prostacyclin (0.1 µg/mL) and apyrase (0.02 U/mL). Platelets were then resuspended in Tyrodes-HEPES buffer containing 2 mmol/L CaCl_2_ and 0.02 U/mL apyrase.

### Platelet life span

To determine platelet life span *in vivo*, mice were injected intravenously with DyLight-488 conjugated anti-GPIX Ig derivative (0.5 µg/g body weight). At 1 h after injection (day 0), as well as at the other indicated time points, 50 µl blood were collected and the percentage of GPIX-positive platelets was determined by flow cytometry.

### Platelet spreading

Cover slips were coated with 100 µg/mL human fibrinogen and blocked with 1% BSA/PBS. After rinsing with Tyrodes-HEPES buffer, washed platelets (100 µL with 0.03×10^6^ platelets/µL) were activated with thrombin (0.01 U/mL) and immediately placed on the coated cover slips. At indicated time points, the cover slips were rinsed again and platelets were visualized with a Zeiss Axiovert 200 inverted microscope (100x/1.4 oil objective). Digital images were recorded using a CoolSNAP-EZ camera (Visitron) and analyzed off-line using Metavue software (Molecular Devises). Four different stages of platelet spreading were evaluated: stage 1 - roundish; stage 2 - filopodia only; stage 3 - filopodia and lamellipodia; stage 4 - fully spread.

### Platelet aggregometry

Light transmission was measured on a Fibrintimer 4 channel aggregometer (APACT Laborgeräte und Analysensysteme) using prp (for ADP) or washed platelets (for all other agonists - 160 µL with 1.56×10^5^ platelets/µL). Measurements in washed platelets were performed in the presence of 70 µg/mL fibrinogen, except for thrombin.

### Flow cytometry

Washed blood was activated with agonists at the indicated concentrations, stained with fluorophore-conjugated monoclonal antibodies at saturating concentrations for 10 minutes at 37°C and analyzed on a FACSCalibur (BD Biosciences). For determination of phosphatidylserine exposure, washed platelets (0.05×10^6^/µL) were stimulated with the indicated agonists in the presence of Annexin-V-DyLight 488 for 15 minutes at 37°C. The reaction was stopped by addition of 500 µl Tyrodes-HEPES containing 3 mM Ca^2+^ and samples were directly analyzed on a FACSCalibur (BD Biosciences).

### Clot retraction

Clot retraction studies were performed at 37°C in an aggregometer tube containing diluted prp (3×10^5^ platelets/µL), thrombin (4 U/mL), and CaCl_2_ (20 mmol/L). Clot retraction was recorded with a digital camera over a time span of 4 hours after activation.

### Western blot analysis

Washed platelets or splenocytes were lysed in lysis buffer (300 mM NaCl, 20 mM TRIS, 2 mM EGTA, 2 mM EDTA, 2 mM Na_3_VO_4_, 10 mM NaF, pH 7.5, supplemented with protease inhibitor cocktail (Sigma-Aldrich) containing 1% Igepal CA-630 for 20 minutes at 4°C, centrifuged at 14,000 rpm for 10 minutes and the supernatants were collected. 4×Laemmli sample buffer was added, and the samples boiled at 95°C for 5 min. Next, samples were separated by sodium dodecyl sulfate polyacrylamide gel electrophoresis (SDS-PAGE) under reducing conditons and blotted onto a polyvinylidene difluoride (PVDF) membrane. The membranes were blocked for 1 hour with 5% BSA in TBS-T at RT and then incubated with the primary antibody at 4°C overnight. The membrane was washed 3×10 minutes in TBS-T, before being incubated with the secondary, HRP-labeled antibody. After extensive washing, the protein was visualized by ECL.

### Platelet adhesion under flow conditions

Heparinized whole blood was perfused over collagen-coated cover slips as described [32]. Before perfusion, anticoagulated blood was incubated with DyLight-488-conjugated anti-GPIX derivative (0.2 µg/mL) at 37°C for 5 minutes. Aggregate formation was visualized with a Zeiss Axiovert 200 inverted microscope (40x/0.60 objective). Phase-contrast and fluorescence pictures were recorded with a CoolSNAP-EZ camera, and analyzed off-line using Metavue software. Thrombus formation was analyzed as the mean percentage of the total area covered by platelets/thrombi in phase contrast images. Mean integrated fluorescence intensity per mm^2^ gave an indication about thrombus volume.

### Intracellular Ca^2+^ measurements

Washed platelets were suspended in Tyrodes-HEPES buffer without Ca^2+^, and loaded with fura-2/AM (5 µmol/L) in the presence of Pluronic F-127 (0.2 µg/mL) for 20 minutes at 37°C. After labeling, platelets were washed once and resuspended in Tyrodes-HEPES buffer containing 1 mmol/L Ca^2+^. Stirred platelets were activated with the respective agonists and fluorescence was measured with an LS 55 fluorimeter (PerkinElmer, USA). Excitation was alternated between 340 and 380 nm, and emission was measured at 509 nm. Each measurement was calibrated using Triton X-100 and EGTA.

### Tail bleeding time

Mice were anaesthetized and 1 mm segment of the tail tip was amputated with a scalpel. Tail bleeding was monitored by gentle absorption of the blood with filter paper at 20 s intervals without making contact with the wound site. When no blood was observed on the paper, bleeding was determined to have ceased. Experiments were stopped after 20 min.

### Intravital microscopy of thrombus formation in FeCl_3_-injured mesenteric arterioles

Intravital microscopy was performed as previously described [33]. In brief, injury of mesenteric arterioles was induced by topical application of 20% FeCl_3_. Adhesion and aggregation of fluorescently labeled platelets in arterioles was monitored for 40 min or until complete occlusion occurred (blood flow stopped for 1 min). Mice were between 4 and 5 weeks of age.

### Mechanical injury of the abdominal aorta

The abdominal cavity of anaesthetized mice was opened to expose the abdominal aorta. An ultrasonic flowprobe (0.5PSB699; Transonic Systems) was placed around the vessel, and thrombus formation was induced by a single firm compression with a forceps upstream of the flowprobe. Blood flow was monitored for 30 min.

### Triple anaesthesia

Mice were anesthetized i.p. with a combination of medetomidine/fentanyl/midazolam (0.5/0.05/5 mg kg^-1^ body weight).

### Statistics

Results from at least three independent experiments per group are presented as mean ± SD. For statistical analysis between experimental groups we applied the unpaired two-tailed student's t-test. All statistical evaluation was done with OriginPro 8.6G. *P*-values<0.05 compared to control were considered statistically significant (*p*-value<0.05 = *; <0.01 = **; <0.001 = ***).

## Results

### EFhd2 is expressed in murine platelets but dispensable for platelet production

**Figure 1:**
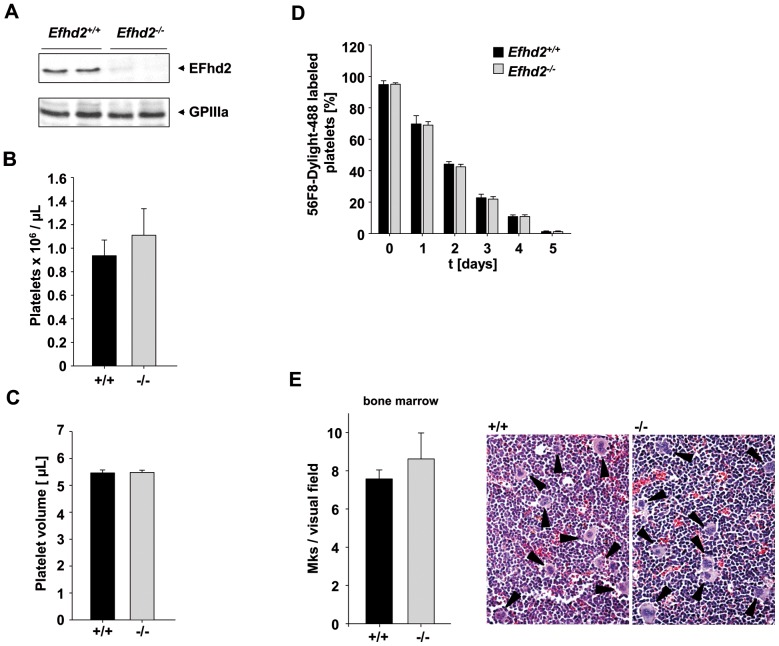
EFhd2 is dispensable for platelet production. (A) Analysis of EFhd2 expression in *Efhd2^+/+^* and *Efhd2^-/-^* platelets by Western blot. Expression of GPIIIa was used as loading control. (B) Peripheral platelet counts and (C) platelet volume of *Efhd2^+/+^* and *Efhd2^-/-^* mice measured with a blood cell counter are depicted. Results are mean ± SD of 7 mice per group. (D) Determination of the platelet life span in wild-type and *Efhd2^-/-^* mice. Mice were injected with a DyLight 488-conjugated anti-GPIX derivate (0.5 µg/g body weight) to label platelets *in vivo*. Results are% of fluorescently labeled platelets at the indicated days after injection as determined by flow cytometry. Values are mean ± SD of 5 mice per group. (E) Determination of MK numbers per visual field (294×221 µm) in hematoxylin and eosin stained BM sections. Values are mean ± SD (n = 5).

To study the role of EFhd2 in platelet physiology, we took advantage of constitutive EFhd2 knockout mice [19]. Western blot analysis confirmed the expression of EFhd2 in wild-type (*Efhd2^+/+^*) platelets and the absence of EFhd2 in platelets from *Efhd2^-/-^* mice (Figure 1 A). Analysis of basic blood parameters with a Sysmex cell counter revealed unaltered white and red blood cell counts as well as unchanged hemoglobin content of red blood cells and haematocrit in *Efhd2^-/-^* mice compared to wild-type controls (Table 1). Platelet count, size, and life span, as well as surface expression levels of prominent glycoprotein receptors were unaltered compared to the wild-type control (Figure 1 B–D, Table 2), indicating that megakaryopoiesis and platelet formation can occur independently of EFhd2. This was corroborated by normal MK numbers in the bone marrow of the mutant animals (wild-type: 7.57±0.5 MK/visual field, *Efhd2^-/-^* 8.6±1.4 MK/visual field; Figure 1 E).

**Table 1:** *Efhd2* ^-/-^ mice display normal hematologic parameters.

Genotype *Efhd2*	WBC×10^3^/µL	RBC×10^6^/µL	HGB [g/dL]	HCT [%]
**+/+**	10.78±3.78	8.39±0.87	14.28±0.80	44.24±3.51
**-/-**	12.95±3.16	8.29±1.40	13.58±2.20	42.98±7.10
***p*** ** value**	n.s.	n.s.	n.s.	n.s

White blood cell count (WBC), red blood cells (RBC), hemoglobin (HGB) and hematocrit (HCT) were determined with a hematologic analyzer (Sysmex) (n = 5 vs. 5, two independent experiments, n.s. = not significant).

**Table 2:** Unaltered expression of platelet glycoproteins in *Efhd2^-/-^* mice.

-	MFI ± SD *Efhd2* ^+/+^	MFI ± SD *Efhd2* ^-/-^	*p* value
**GPIb**	330.6±13.4	329.8±8.3	n.s.
**GPVI**	41.0±1.0	39.4±0.9	n.s.
**GPV**	242.0±4.8	242.6±3.3	n.s.
**GPXI**	377.4±5.5	380±8.6	n.s.
**CD9**	968.2±9.3	969.6±17.0	n.s.
**αIIbβ3**	543.6±17.9	555.8±19.5	n.s.
**α2**	40.4±1.1	40.6±1.1	n.s.
**β1**	102.4±5.2	101.6±2.3	n.s.
**CLEC-2**	101.8±13.5	100.6±2.6	n.s.

Expression of glycoproteins on the platelet surface was determined by flow cytometry. Diluted whole blood from the indicated mice was incubated with FITC-labeled antibodies at saturating conditions for 15 minutes at RT, and platelets were analyzed directly. Data are expressed as mean fluorescence intensity ± SD (n = 4) and are representative of 3 individual experiments. n.s. = not significant, SD = standard deviation.

### Platelet activation and aggregation were unaffected by loss of EFhd2

**Figure 2:**
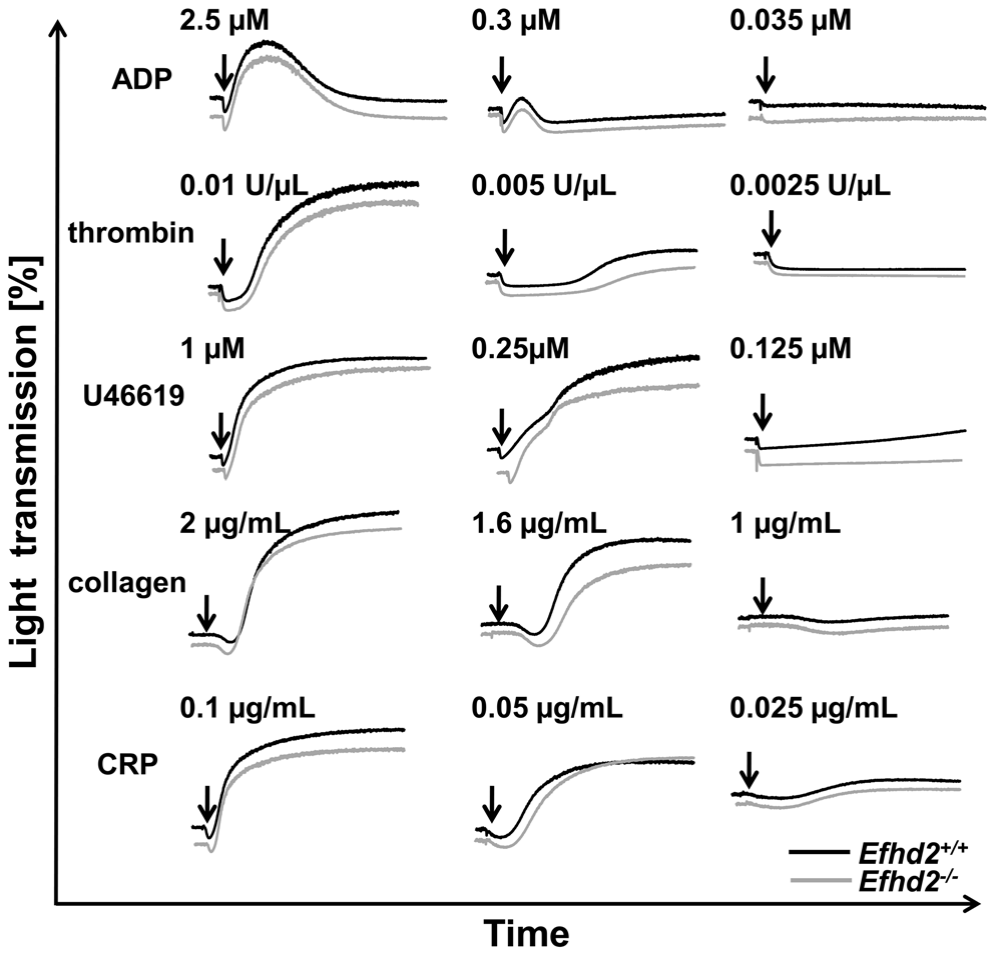
Unaltered aggregation response of *Efhd2^-/-^* platelets. Washed platelets from *Efhd2^+/+^* (black line) and *Efhd2^-/-^* (gray line) mice were activated with the indicated agonist concentrations and light transmission was recorded on a Fibrintimer 4-channel aggregometer. ADP measurements were performed in prp. Representative aggregation traces of at least 3 individual experiments are depicted.

**Figure 3:**
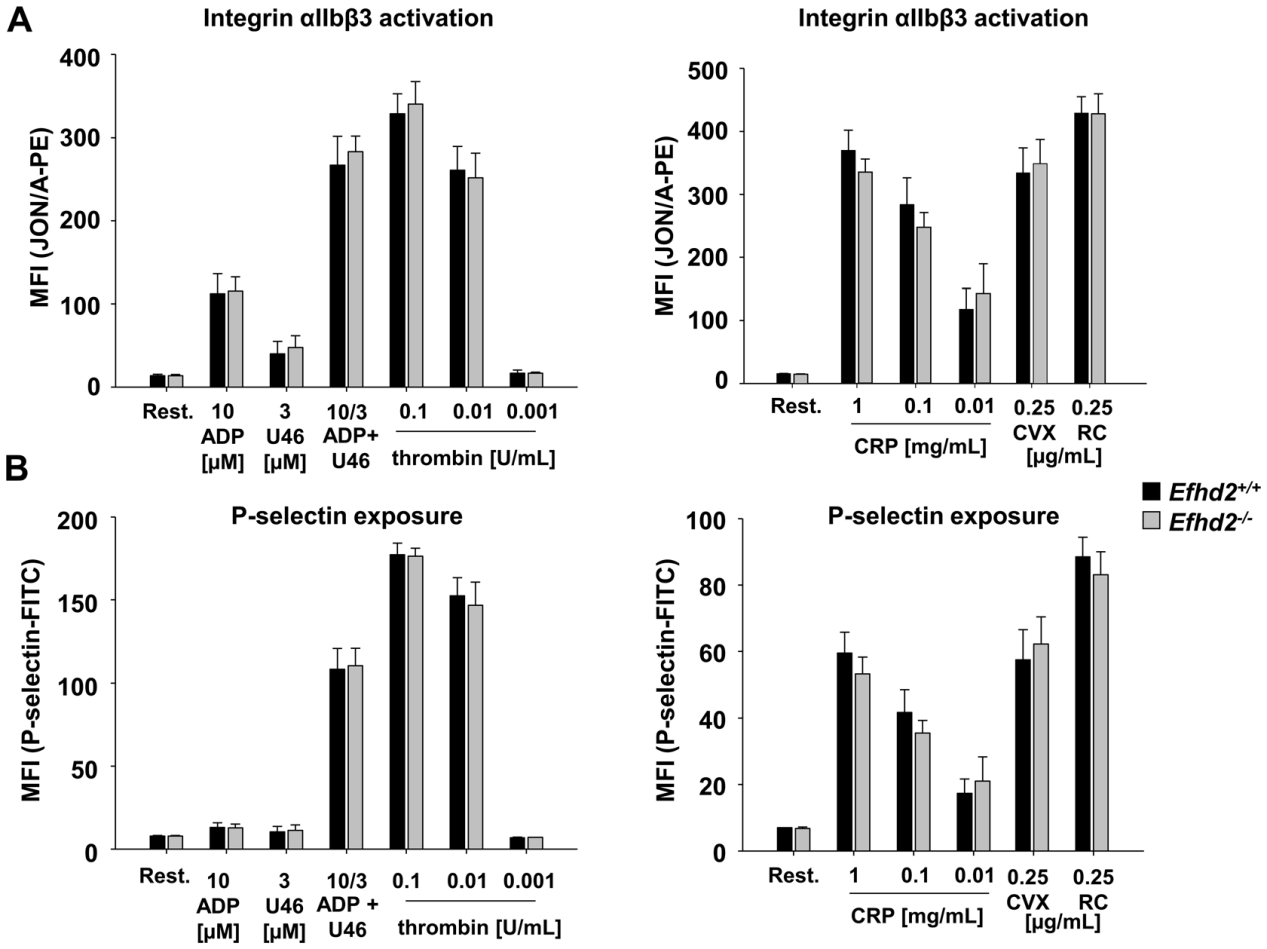
Normal αIIbβ3 activation and α-granule release in *Efhd2^-/-^* platelets. Flow cytometric analysis of integrin αIIbβ3 activation (A) and degranulation-dependent P-selectin exposure (B) in response to the indicated agonists in *Efhd2^+/+^* and *Efhd2^-/-^* platelets. Results are mean fluorescence intensities (MFI) ± SD of 4 mice per group and are representative of 4 individual experiments. CRP: collagen-related peptide, CVX: convulxin, and RC: rhodocytin.

To study the consequences of EFhd2-deficiency on platelet function, we performed *ex vivo* aggregation studies. *Efhd2^-/-^* platelets showed unaltered shape change and aggregated normally in response to the G protein-coupled receptor (GPCR) agonists thrombin, ADP and the thromboxane A_2_ (TxA_2_) analogue U46619 as well as after stimulation of the immunoreceptor tyrosine-based activation motif (ITAM)-coupled receptor glycoprotein (GP) VI by collagen or CRP (Figure 2). These findings were confirmed by flow cytometric analysis of integrin αIIbβ3 activation (Figure 3 A) and of degranulation-dependent P-selectin surface exposure (Figure 3 B). Similarly, platelet activation via the (hem)ITAM-receptor CLEC-2 by the snake venom toxin rhodocytin (RC) was unaltered in *Efhd2^-/-^* platelets (Figure 3). These results demonstrate that EFhd2 is dispensable for platelet activation by classic agonists.

### *Efhd2^-/-^* platelets show normal integrin outside-in signaling

**Figure 4:**
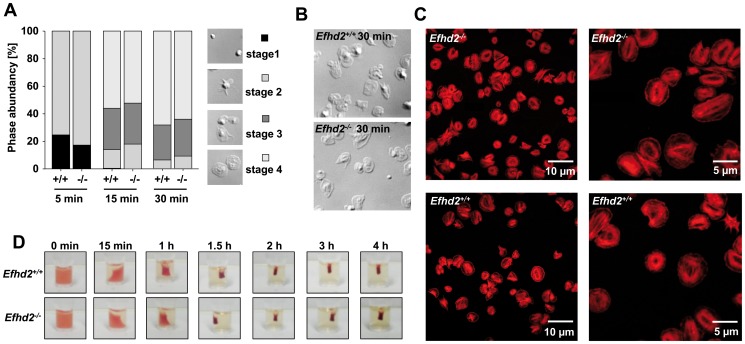
Normal integrin outside-in signaling in *Efhd2^-/-^* platelets. (A-C) Washed platelets of *Efhd2^+/+^* and *Efhd2^-/-^* mice were allowed to spread on fibrinogen (100 µg/mL) for 30 min after stimulation with 0.01 U/mL thrombin. (A) Statistical evaluation of the percentage of spread platelets at different spreading stages and (B) representative differential interference contrast (DIC) images of 2 individual experiments. 1: roundish, 2: only filopodia, 3: filopodia and lamellipodia, 4: fully spread. (C) Visualization of filamentous actin (red) in spread (30 min) *Efhd2^+/+^* and *Efhd2^-/-^* platelets by cofocal microscopy. (D) Clot retraction of prp upon activation with 4 U/mL thrombin in the presence of 20 mmol/L CaCl_2_ at the indicated time points (n = 6).

Ligand-occupied integrin αIIbβ3 mediates outside-in signaling, leading to cytoskeletal reorganization and platelet spreading [34]. EFhd2 has been proposed to play a crucial role in these processes in different cell types [18]. To test this in platelets, *Efhd2^-/-^* and wild-type platelets were allowed to spread on a fibrinogen-coated surface in the presence of low concentrations of thrombin (Figure 4 A). Surprisingly, *Efhd2^-/-^* and wild-type platelets formed filopodia and lamellipodia with similar kinetics and after 30 minutes the number of fully spread platelets was comparable between the two groups (Figure 4 A and B). In line with this, *Efhd2^-/-^* spread platelets displayed normal actin distribution and content (Figure 4 C). Integrin αIIbβ3 outside-in signaling also regulates clot retraction [35]. Therefore, we induced clot formation in platelet rich plasma (prp) by addition of a high dose of thrombin (4 U/ml) and 20 mmol/L CaCl_2_, and monitored retraction over time. No differences between wild-type and *Efhd2^-/-^* platelets were observed (Figure 4 D). Together, our data demonstrate that EFhd2 is not required for the orchestration of actin rearrangements during integrin αIIbβ3-controlled spreading and clot retraction.

### *Efhd2^-/-^* platelets show normal Ca^2+^ mobilization and slightly increased coagulant activity

**Figure 5:**
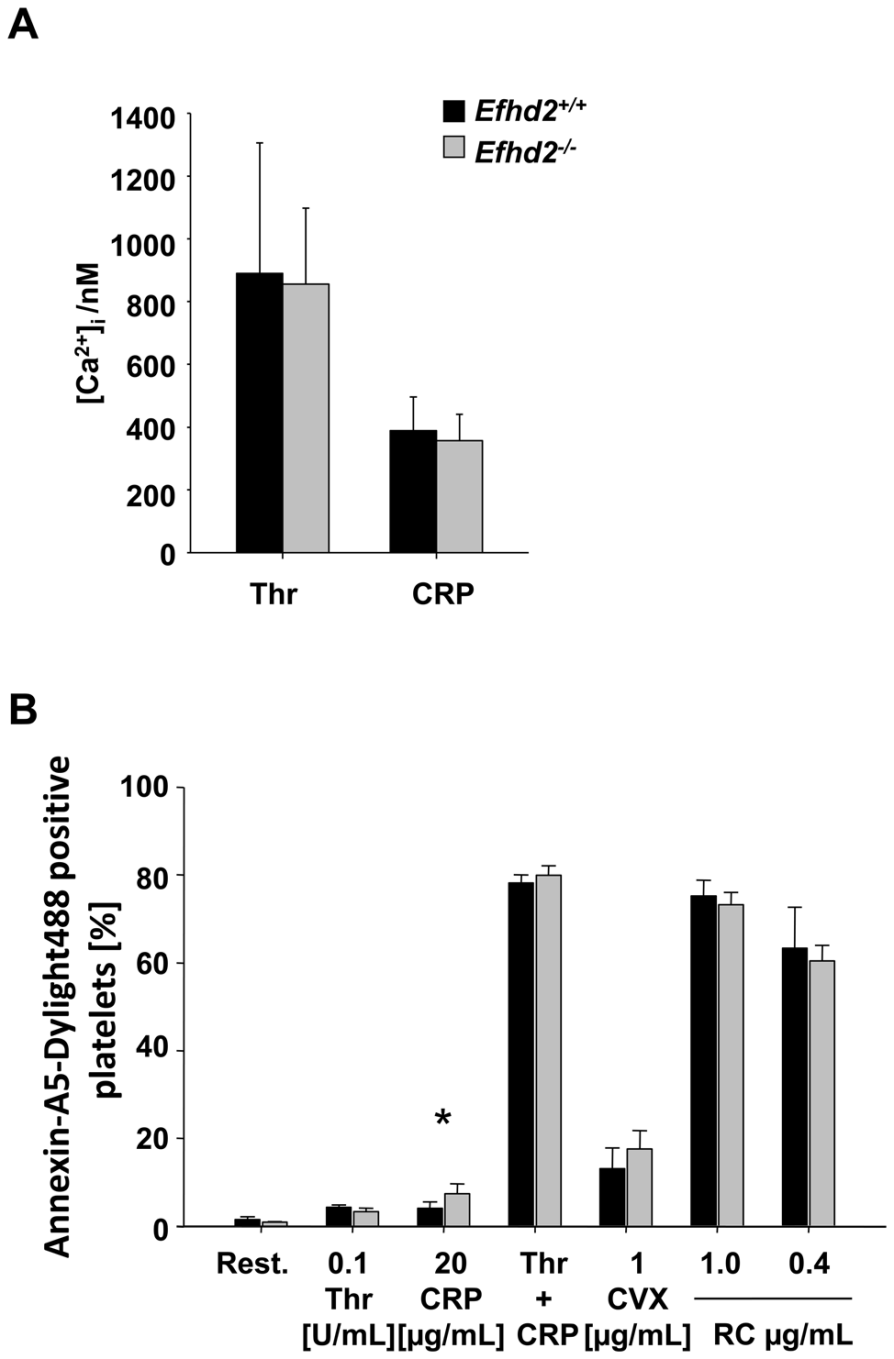
Normal Ca^2+^-mobilization but slightly increased procoagulant activity in EFhd2-deficient platelets upon stimulation. (A) Maximal increase of cytosolic Ca^2+^ concentration ([Ca^2+^]_i_) of *Efhd2^+/+^* (black bars) and *Efhd2^-/-^* platelets (gray bars) after activation with the indicated agonists (thrombin 0.1 U/ml, CRP 4 µg/ml). (B) Flow cytometric analysis of phosphatidylserine (PS) exposure in response to the indicated agonists in *Efhd2^+/+^* and *Efhd2^-/-^* platelets. Washed platelets were stained with Annexin-V-DyLight-488 in the presence of Tyrodes-HEPES buffer containing 3 mmol/L Ca^2+^. The values represent the mean fluorescence intensity (MFI) ± SD for 5 mice per group in 3 independent experiments. Thr: thrombin, CRP: collagen related peptide, CVX: convulxin, RC: rhodocytin, n.s.: not significant, *p<0.05.

Agonist-induced platelet activation leads to an increase in cytosolic calcium concentrations ([Ca^2+^]_i_) through release of Ca^2+^ from intracellular stores and Ca^2+^ entry across plasma membrane Ca^2+^ channels [7]. EFhd2 possesses two functional EF hand domains capable of binding Ca^2+^ thereby mediating self-oligomerization and modulating F-actin bundling and cell spreading [18]. To test whether the lack of EFhd2 affected Ca^2+^ signaling in platelets, we studied agonist-induced changes in [Ca^2+^]_i_ fluorimetrically. *Efhd2^-/-^* platelets displayed normal Ca^2+^ store release and entry in response to CRP and thrombin (Figure 5 A) indicating that EFhd2 is dispensable for Ca^2+^ signaling in platelets after stimulation with major platelet agonists.

GPVI-stimulated platelets facilitate coagulation by the exposure of negatively charged phosphatidylserine (PS) on their outer surface, thereby providing high affinity binding sites for key coagulation factors [36]. To test a possible role of EFhd2 in the induction of coagulant activity, washed platelets were stimulated with different agonists and PS exposure was analyzed. After stimulation with the combination of CRP/thrombin as well as high concentrations of rhodocytin the majority of wild-type and *Efhd2^-/-^* platelets exposed PS on their surface (Figure 5 B). Interestingly, *Efhd2^-/-^* platelets displayed a slightly increased PS exposure in response to CRP which produced only a minor increase in PS exposure in wild-type platelets (Figure 5 B). These results revealed that EFhd2 might to a minor extent inhibit ITAM-induced coagulant activity in platelets.

### Unaltered aggregate formation of *Efhd2^-/-^* platelets on collagen under flow

**Figure 6:**
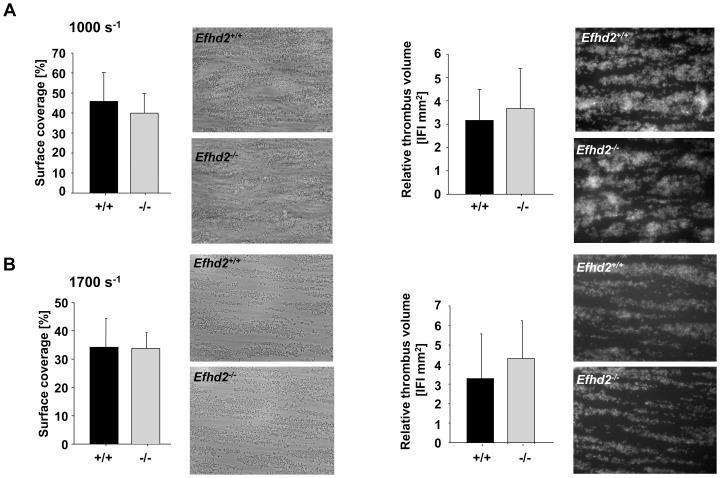
Normal adhesion and aggregate formation of *Efhd2^-/-^* platelets on collagen under flow. Whole blood from *Efhd2^+/+^* or *Efhd2^-/-^* mice was perfused over a collagen-coated surface (0.2 mg/mL) at a shear rate of 1000 s^−1^ (A) or 1700 s^−1^ (B). Representative images of aggregate formation on collagen after 4 minutes of perfusion time. Mean surface coverage (left) and relative thrombus volume expressed as integrated fluorescence intensity (IFI) (right) ± SD of 5 *Efhd2^+/+^* and 5 *Efhd2^-/-^* mice.

Thrombus formation at sites of vascular injury requires stable shear-resistant platelet adhesion on the ECM as well as auto- and paracrine platelet activation by locally released or generated secondary mediators [37]. To address the effect of EFhd2-deficiency on these processes, platelet adhesion to collagen was studied in an *ex vivo* whole blood perfusion system. When blood was perfused over immobilized collagen at a shear rate of 1000 s^−1^ or 1700 s^−1^ wild-type and *Efhd2^-/-^* platelets rapidly adhered to the collagen surface with comparable kinetics and recruited additional platelets from the blood stream resulting in the formation of stable three-dimensional thrombi (Figure 6). As a result, both the surface area covered by platelets (1000 s^−1^: *Efhd2^+/+^*: 44.8±14.4% vs. *Efhd2^-/-^*: 39.9±9.8%; 1700 s^−1^: *Efhd2^+/+^*: 34.2±10.2% vs. *Efhd2^-/-^*: 33.7±5.6%; Figure 6 A and B, left) and the thrombus volume (1000 s^−1^: *Efhd2^+/+^*: 3.2±1.3 vs. *Efhd2^-/-^*: 3.7±1.7; 1700 s^−1^: *Efhd2^+/+^*: 3.3±2.3 vs. *Efhd2^-/-^*: 4.4±1.9 Figure 6 A and B, right) at the end of the perfusion period were similar in wild-type and *Efhd2^-/-^* platelets. These results demonstrate that EFhd2 is not essential for platelet adhesion and aggregate formation on collagen under flow.

### Normal hemostasis and arterial thrombus formation in *Efhd2^-/-^* mice

**Figure 7:**
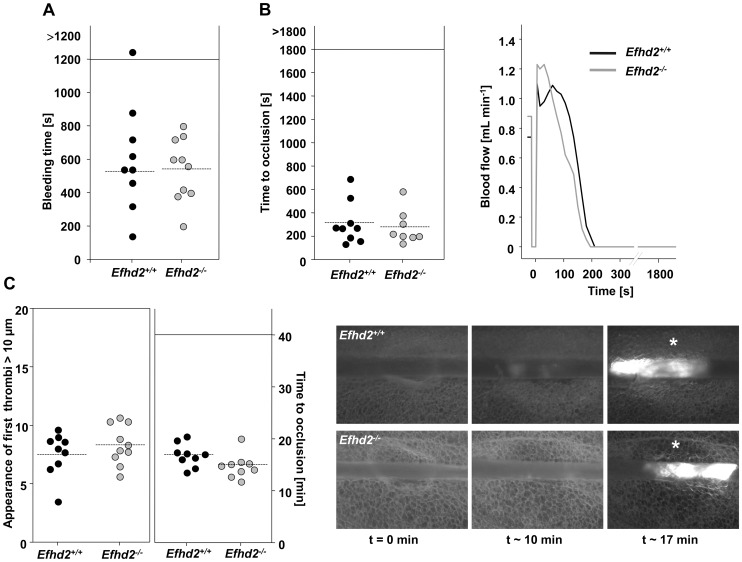
Unaltered thrombotic and hemostatic function in EFhd2-deficient mice. (A) Tail bleeding times of *Efhd2^+/+^* and *Efhd2^-/-^* mice. Each symbol represents one animal. (B) Time to stable vessel occlusion of *Efhd2^+/+^* and *Efhd2^-/-^* mice. The abdominal aorta was injured by firm compression with a forceps and blood flow was monitored for 30 min. Each symbol represents one animal. (C) Appearance of first thrombi (left) and time to occlusion (middle). Small mesenteric arterioles were injured by topical application of FeCl_3_ and thrombus formation of fluorescently labeled platelets was monitored using intravital microscopy. Each symbol represents one mesenteric arteriole. The horizontal dotted line indicates the mean time to vessel occlusion. Representative images of the FeCl_3_-induced injury model of mesenteric arterioles in *Efhd2^+/+^* and *Efhd2* ^-/-^ mice, asterisk indicates stable occlusion of the vessel (right).

To study the effect of EFhd2-deficiency on hemostasis, tail bleeding times were determined (Figure 7 A). *Efhd2^-/-^* and wild-type mice exhibited comparable bleeding times (mean bleeding time: *Efhd2^+/+^*: 527±229 s vs. *Efhd2^-/-^*: 542±189 s) suggesting that hemostasis is not affected in the absence of EFhd2.

To examine whether EFhd2-deficiency affected thrombus formation *in vivo*, we subjected the mice to a thrombosis model where the abdominal aorta is mechanically injured and blood flow is monitored with an ultrasonic perivascular Doppler flow probe. In line with our *in vitro* results, wild-type and *Efhd2^-/-^* mice formed occlusive thrombi with similar kinetics (mean occlusion time: *Efhd2^+/+^*: 315±182 s vs. *Efhd2^-/-^*: 279±144 s; Figure 7 B). Similar results were obtained in a model of FeCl_3_-induced injury of mesenteric arterioles. The appearance of first thrombi>10 µm (*Efhd2^+/+^*: 7.58±1.87 min vs. *Efhd2^-/-^*: 8.36±1.70 min; Figure 7 C, left) as well as the time to vessel occlusion (*Efhd2^+/+^*: 16.89±2.23 min vs. *Efhd2^-/-^*: 15.51±2.75 min; Figure 7 C, middle) was comparable between wild-type and mutant mice. Representative images of thrombus formation in this model are shown in Figure 7C, right. These results indicate that EFhd2 is not required for hemostatic and thrombotic function of platelets *in vivo*.

### Normal expression of cytoskeletal and signaling proteins

**Figure 8:**
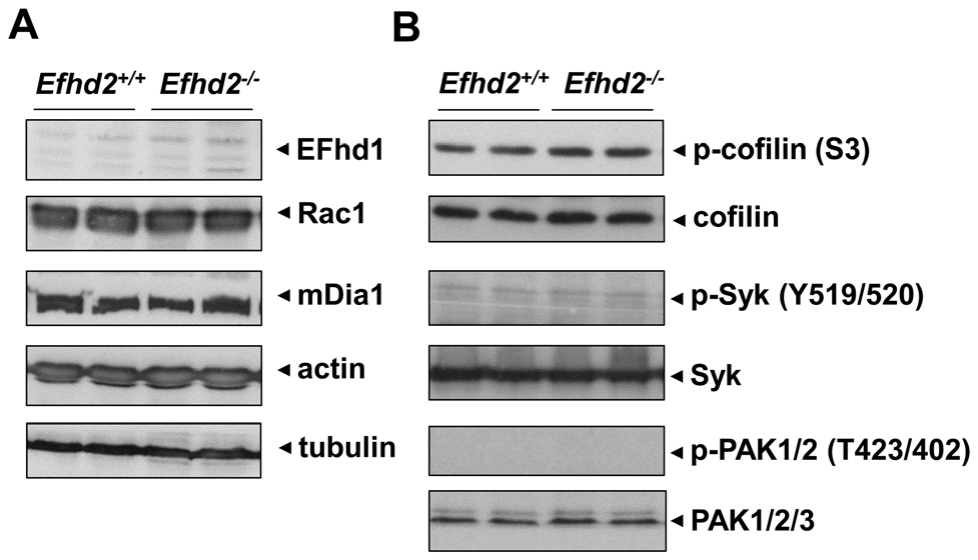
Normal expression levels of specific proteins in EFhd2-deficient platelets. (A) Analysis of EFhd1, Rac1, mDia1, actin and tubulin expression in *Efhd2^+/+^* and *Efhd2^-/-^* platelets by Western blot. (B) Analysis of phospho-cofilin, phospho-Syk, and phospho-PAK1/2 expression in *Efhd2^+/+^* and *Efhd2^-/-^* platelets by Western blot. Staining of the respective non-phosphorylated proteins served as loading controls.

EFhd1 is a close homologue of EFhd2 and exhibits a high degree of sequence identity at the protein level (64.58%) [15]. To test a possible compensatory up-regulation of EFhd1 expression in *Efhd2^-/-^* platelets, we performed Western blot analysis and found the protein to be absent in both wild-type and *Efhd2^-/-^* platelets (Figure 8 A). In addition, actin and tubulin content and the expression of Rac1 and mDia1 were unaltered in *Efhd2^-/-^* platelets (Figure 8 A). Furthermore, we detected comparable levels of phosphorylated (inactive) cofilin in mutant and wild-type platelets and did not observe compensatory basal activation of Syk or PAK1/2 (Figure 8 B) in resting platelets.

## Discussion

In this study, constitutive knockout mice for EFhd2 were used to assess the role of this Ca^2+^- binding cytoskeletal adaptor protein for platelet function *in vitro* and *in vivo*. We show that activation responses are comparable between EFhd2-deficient and wild-type platelets *in vitro* and that hemostasis and arterial thrombosis are unaltered *in vivo*, indicating that EFhd2 is not required for hemostatic platelet function in mice.

Rearrangements of the platelet actin and tubulin cytoskeleton are essential for platelet production, but also for proper platelet function and hemostasis. These processes are regulated by a variety of cytoskeletal proteins including Rho GTPases [38]–[41] and actin-binding proteins, such as cofillin and ADF [39]. Interestingly, EFhd2 was described in different cell types as an F-actin binding protein, which regulates actin remodeling in a Ca^2+^- and Rac1-dependent manner, as well as cell spreading and the accessibility of F-actin to cofilin [18], [23]–[26]. However, due to the lack of an appropriate animal model, these studies were so far only performed in cell culture systems with the help of recombinant EFhd2 proteins carrying various mutations or gene silencing. In contrast, we have recently demonstrated that EFhd2 is not involved in the regulation of the total F-actin content in B cells [19]. In line with this, we here show that *Efhd2^-/-^* platelets exhibit an unaltered F-actin content (Figure 4 C and 8 A) associated with unaltered spreading (Figure 4 A–C) and clot retraction (Figure 4 D), demonstrating that EFhd2 is not essential for orchestrating actin rearrangements in murine platelets. Importantly, the close homolog EFhd1 was not compensatory up-regulated in EFhd2-deficient platelets (Figure 8 A). Furthermore, neither Rac1 expression nor cofilin (de-)phosphorylation were altered in resting *Efhd2^-/-^* platelets compared to wild-type control (Figure 8 A and B) and also tubulin and the RhoA effector mDia1, which is known to regulate actin polymerization and microtubule stabilization, were normally expressed, indicating that EFhd2-deficiency has no major impact on the expression and activity of important cytoskeleton regulating proteins. Additionally, we found also normal platelet count, size, glycoprotein expression, platelet life span and MK number in the bone marrow (Figure 1), suggesting that EFhd2 is dispensable for platelet production *in vivo*.

Besides the ability of EFhd2 to interact with actin or actin-binding molecules, EFhd2 contains two functional EF-hand domains capable of binding Ca^2+^ [17]. Furthermore, EFhd2 is involved in B cell receptor-mediated apoptosis by amplifying Ca^2+^ influx through positive regulation of Syk [16]. However, we found normal platelet activation downstream of GPCRs as well as (hem)ITAM-bearing receptors (Figure 2 and 3) and unaltered Ca^2+^-mobilization after platelet stimulation (Figure 5 A), demonstrating that EFhd2 is not required for these processes.

Additionally, expression of Syk and PAK1/2/3, as well as their phosphorylation were unaltered in *Efhd2^-/-^* platelets under resting conditions compared to wild-type control (Figure 8 B). Nevertheless, coagulant activity was slightly but significantly increased specifically after GPVI stimulation (Figure 5 B). The physiological significance of this difference is unclear, but may imply that EFhd2 negatively regulates GPVI-triggered procoagulant activity. However, we found unaltered platelet adhesion and aggregate formation under flow on collagen in the absence of EFhd2 (Figure 6), and, therefore, do not support this conclusion. In line with these *in vitro* findings, our *in vivo* analysis of EFhd2-deficient mice demonstrated normal hemostasis and arterial thrombosis (Figure 7), indicating that EFhd2 is dispensable for platelet function in models of hemostasis and thrombosis.

Taken together, our results demonstrate that EFhd2 is not required for proper platelet production and function of the cells in hemostasis and thrombosis in mice. Most likely, redundancies with other Ca^2+^- and actin-binding proteins can compensate for the loss of EFhd2. Given that EFhd2 expression in platelets is up-regulated during sepsis and has been associated with neurodegenerative processes, it is tempting to speculate that EFhd2 may be relevant for platelet functions other than clot formation such as inflammation, immune defense or wound healing. This will require further investigation and we believe that EFhd2-deficient mice will be a valuable tool for these studies.
